# Context-Dependent Differences in Muscle Architecture Following Fatigue in Ultramarathon Athletes: A Comparison Between Laboratory and Real Race Settings

**DOI:** 10.3390/diagnostics16071080

**Published:** 2026-04-02

**Authors:** Juan Vicente-Mampel, Ignacio Martinez-Navarro, Eladio Collado, Raúl Lopez-Grueso, Eloy Jaenada-Carrilero, Carlos Hernando

**Affiliations:** 1 Department of Physiotherapy, Medicine and Health Science School, Catholic University of Valencia, 46090 Torrent, Spain; eloy.jaenada@ucv.es; 2 Physical Education and Sports Department, University of Valencia, 46010 Valencia, Spain; ignacio.martinez-navarro@uv.es; 3 Faculty of Health Sciences, Jaume I University, 12071 Castellon, Spain; colladoe@uji.es; 4 Department of Education and Specific Didactics, Jaume I University, 12071 Castellon, Spain; grueso@uji.es (R.L.-G.); hernando@uji.es (C.H.); 5 Sport Service, Jaume I University, 12071 Castellon, Spain

**Keywords:** muscle fatigue, ultrasonography, running

## Abstract

**Background/Objectives**: Understanding how different fatigue contexts influence muscle architecture is essential for optimizing training and recovery strategies in endurance athletes. Ultramarathon running involves prolonged mechanical load and high eccentric demands, which may elicit different acute responses compared to controlled laboratory protocols. This study aimed to examine the effects of time, condition (laboratory vs. race), and muscle on ultrasound-derived muscle architecture in ultratrail runners. **Methods**: A repeated-measures within-subject design was employed. Forty ultratrail runners completed two fatigue conditions: (1) a standardized laboratory downhill running protocol and (2) an ultramarathon race (CSP 2025; 106 km, +5600 m elevation gain). Muscle thickness and pennation angle of the rectus femoris, vastus lateralis, and medial gastrocnemius were assessed using ultrasound before and after each condition. Linear mixed models were used to evaluate the effects of time, condition, muscle, and their interactions. **Results**: Forty participants were recruited; 29 completed all assessments. No significant effects of time or condition were observed for muscle thickness, and no interaction effects were detected, indicating that muscle size remained stable across conditions and time points. A significant main effect of muscle was identified (*p* < 0.001), reflecting inherent morphological differences, with greater thickness in the vastus lateralis compared to the rectus femoris and medial gastrocnemius. In contrast, pennation angle showed a significant main effect of condition (*p* = 0.031) and a significant condition × muscle interaction (*p* = 0.005), indicating muscle-specific differences between laboratory and race contexts. No significant effect of time was observed for pennation angle. **Conclusions**: Muscle thickness appears to remain stable following acute fatigue, regardless of the assessment context. In contrast, pennation angle may be more sensitive to condition-specific and muscle-dependent factors. These findings suggest that ultrasound-derived architectural changes observed immediately after exercise likely reflect acute physiological responses rather than true structural adaptations. Therefore, the interpretation of muscle architecture should consider both contextual factors and methodological constraints.

## 1. Introduction

Recently, specific assessment techniques have been implemented to evaluate the architectural characteristics of the quadriceps (vastus lateralis and rectus femoris) and medial gastrocnemius muscles, with the aim of improving the diagnosis, prognosis, and performance monitoring in endurance athletes. Consequently, the precise evaluation of these architectural features has become an important tool in sports medicine, even in injury prevention programs. In fact, ultrasound and other noninvasive imaging techniques have substantially enhanced our understanding of muscle structure–function relationships, including implications for injury risk and performance [1]. For example, the measurement of muscle fascicle length (FL) via ultrasound has become a widely accepted method for detecting increases associated with training, as well as reductions related to muscle disuse or aging [2]. Recent studies have shown that muscle morphological adaptations to training in the vastus lateralis, rectus femoris, and medial gastrocnemius vary greatly between individuals [3]. These inter-individual differences highlight the importance of individualized monitoring to optimize training outcomes. Furthermore, these data suggest that variations in muscle architecture may influence the behavior of active muscle components and contribute to differences in force generation, fatigue resistance, and injury susceptibility [4].

Given the importance of muscle size and architecture in the muscles actually assessed, it is essential to consider intrinsic muscle properties, such as fiber type composition and fascicle length, which significantly influence maximal power output and energy expenditure [5]. Muscle activation and strength development have been shown to differ across exercises targeting the quadriceps and gastrocnemius muscles, influencing adaptations in fascicle length, pennation angle, and muscle volume [6]. These findings contribute to the refinement of musculoskeletal models that aim to predict mechanical muscle output during contraction more accurately [7]. Additionally, fiber type and fascicle length account for substantial variability in maximal power output and energy cost, underscoring the importance of fascicle length in the power–economy trade-off [8]. Ultramarathon races have grown exponentially in recent years; in this context, ultramarathon runners are exposed to unstable environmental and weather conditions for prolonged periods, as well as constant changes in terrain topography and altitude, with significant positive and negative elevation gains [9].

Such prolonged and demanding conditions can lead to temporary muscular fatigue, either due to the accumulation of metabolites within the intracellular space or depletion of energy-producing substrates [10]. Specifically, the challenging terrain in ultramarathon running induces intense muscular activity, particularly during the eccentric contraction phase, resulting in muscle damage, hypercatabolism, elevated inflammatory biomarkers, and oxidative stress [11]. Consequently, muscle damage contributes to deficits in strength, rate of force development, and power output [12]. Additionally, exercise-induced fatigue exacerbates these impairments through fluid redistribution (muscle swelling) and alterations in neuromuscular activation, thereby disrupting key regulatory mechanisms [13]. Importantly, these acute responses may reflect transient edema or fluid shifts rather than true long-term architectural adaptations, which must be considered when interpreting post-exercise measurements. However, markers of muscle architecture and composition assessed via ultrasound did not show significant adaptation following the specific training intervention described in the cited study [14]. Notably, in the medial gastrocnemius muscle, eccentric contractions (rather than active stretching) appear to induce greater muscle damage than prolonged contractions at extended lengths [15]. These findings highlight the intricate nature of muscle responses to fatigue and mechanical stress, particularly among endurance athletes who encounter prolonged and variable conditions, such as those experienced in ultramarathon events. Consequently, comprehending the subtleties of muscle architectural adaptations in response to diverse fatigue protocols is crucial for optimizing performance and recovery strategies within this population.

Despite these advances, previous research has methodological limitations, including the absence of direct comparisons between controlled laboratory protocols and real competition settings, limited use of statistical approaches capable of handling repeated measures and inter-individual variability, and reduced ecological validity in experimental designs. Importantly, there is a lack of studies directly comparing controlled laboratory fatigue protocols (e.g., downhill running) with real ultramarathon competitions regarding their impact on the architecture of the quadriceps and medial gastrocnemius, particularly in the context of fatigue and performance [16]. Furthermore, individual variability, prior lower extremity injuries, or neuromuscular conditions may influence ultrasound assessments, highlighting the need for standardized evaluation protocols [17,18]. The objective of this study was to investigate the effects of time (pre vs. post), condition (laboratory vs. race), and muscle on muscle architecture, specifically focusing on muscle thickness and pennation angle, and to ascertain whether these factors interact within a repeated-measures framework. Furthermore, this study aimed to explore the potential impact of the assessment context on muscle architectural properties by evaluating the stability of muscle thickness and the sensitivity of pennation angle to condition-specific factors across different muscles.

## 2. Materials and Methods

### 2.1. Study Design

This study employed a repeated-measures within-subject design, in which participants were assessed under two experimental conditions (laboratory versus race) and at two time points (pre- and post-exercise). This methodological approach facilitated the investigation of within-subject alterations in muscle architecture while accounting for inter-individual variability. Given the absence of randomization and the ecological nature of the race condition, this study is more accurately categorized as a quasi-experimental design rather than a true controlled experiment. This study received approval from the Ethics Committee of Universitat Jaume I (Castellón) (reference number CEISH/103/2024). Prior to participation, all participants provided informed consent in accordance with the ethical standards of the Declaration of Helsinki [19]. The trial adhered to the STROBE guidelines [20]. Additionally, the study protocol was prospectively registered in the official ClinicalTrials.gov registry (ID: NCT06969898).

### 2.2. Participants

The present study involved ultra-trail runners registered as official participants in the CSP 2025 race, which was conducted in Castellón, Spain, on 11–12 April 2025. A total of 40 participants, comprising both men and women, were included. The study population consisted of healthy adult runners aged between 18 and 60 years who satisfied specific eligibility criteria. Recruitment and data collection were conducted during the pre-race period, with eligible participants being contacted and assessed prior to the event. The inclusion criteria mandated that participants be healthy adults aged between 18 and 60 years with prior experience in ultra-endurance events. Specifically, all participants were required to have completed at least two ultra-trail races of 65 km or longer and to be officially registered as participants in the CSP 2025 race (Castellón, Spain). The exclusion criteria encompassed a history of heart or kidney disease and the ongoing use of any medication at the time of recruitment.

### 2.3. Experimental Design

All participants underwent two fatigue-inducing conditions: a controlled laboratory downhill running protocol and an actual ultramarathon race, with assessments conducted both prior to and following each condition, allowing comparison between controlled and real-world fatigue scenarios.

#### 2.3.1. Fatigue-Inducing Protocol Under Controlled Conditions (Laboratory)

A controlled laboratory protocol was developed to induce neuromuscular fatigue through a standardized downhill running (DR) session. The first visit, conducted 4–6 weeks prior to an ultramarathon race, involved baseline measurements, including blood sampling, muscle morphology assessment via ultrasound, isometric strength tests, and an uphill walking economy test. Participants then engaged in the DR protocol, which entailed 5 km of treadmill running at a 15% decline, designed to replicate descents typical of major ultratrail races [16]. The running speed was individualized to match the heart rate corresponding to the first ventilatory threshold (VT1) obtained during the uphill CPET, ensuring consistent relative intensity [21]. Mechanical variables, such as step length, step frequency, ground contact time, and vertical oscillation, were recorded using a foot-mounted power meter [22,23]. This protocol enabled the consistent induction of fatigue within a controlled setting, replicating the mechanical and physiological demands associated with trail running descents. Muscle architecture was evaluated in the laboratory both before and following the participants’ completion of a 5 km downhill running protocol.

#### 2.3.2. Fatigue Assessment During Real Competition: Ultramarathon Race Protocol (Race)

The field-based protocol was implemented during the 13th edition of the CSP (Castelló–Penyagolosa), which took place in Castellón, Spain, on 12 April 2025, commencing at 00:00 a.m. from the athletics track at Universitat Jaume I. The course encompassed a total distance of 106 km, featuring a positive elevation gain of 5600 m and a negative elevation of 4400 m. Participants were allotted a maximum time of 25 h and 30 min to complete the race. This authentic ultratrail competition offered an ecologically valid setting for evaluating neuromuscular fatigue and physiological adaptations under prolonged, high-demand endurance conditions. Additional information about the event can be found at: https://www.penyagolosatrails.com/csp/ (accessed on 7 July 2025). Pre-race assessments were performed 6–12 h prior to the race using the same ultrasound device and assessor as in the laboratory. Pre-race assessments were performed 6–12 h prior to the race, using the same ultrasound device and assessor as in the laboratory. A temperature-controlled tent and portable treatment table were used to ensure reproducibility. At race completion, runners were guided directly to a second mobile testing area located at the finish line, where post-race ultrasound assessments were performed within 2–4 min of finishing. The same assessor performed all measurements to eliminate inter-rater variability.

### 2.4. Outcome Measures and Muscle Architecture Assessment

#### 2.4.1. Measurement of Anthropometric Variables

At the onset of the study, the researchers conducted an initial interview to gather anthropometric measurements from the participants. Weight and height were measured directly using a calibrated digital scale (Seca 813) and stadiometer (Seca 217), respectively. BMI was calculated as kg/m^2^, incorporating the age and sex of the study participants.

#### 2.4.2. Primary Outcome: Muscle Architecture Assessed Using Ultrasound

Ultrasound images were acquired using a Philips Lumify^®^ portable ultrasound system with an L12-4 linear transducer (Philips Healthcare, Best, The Netherlands). Depth, gain, and dynamic range were standardized across sessions. Two architectural features were assessed: pennation angle (PA) and muscle thickness (MT) in the rectus femoris, vastus lateralis, and medial gastrocnemius. Image analysis was performed using the ImageJ DICOM viewer (Pixmeo) and ImageJ (NIH) software (Version 1.53, National Institutes of Health, Bethesda, MD, USA) [24,25]. In both measurement sessions, a generous amount of ultrasound gel was applied to minimize pressure on the skin. Images were obtained by maintaining the transducer in a vertical position relative to the skin. The mean of the three measurements was used [26]. The measurement sites were pre-marked with a surgical skin marker [27].

##### Medial Gastrocnemius

To assess the medial gastrocnemius muscle, participants were positioned in a prone posture with the knee fully extended. While the participants remained relaxed, longitudinal ultrasound measurements were conducted parallel to the orientation of the muscle fibers within the medial gastrocnemius belly. The measurement site was identified at one-third of the distance from the center of the knee joint to the calcaneus, measured proximally along the length of the leg [28]. Participants maintained passive muscle relaxation during imaging to avoid fascicle distortion.

##### Vastus Lateralis and Rectus Femoris

Ultrasound imaging of the vastus lateralis (VL) and rectus femoris (RF) was performed to evaluate muscle thickness and pennation angle in the dominant leg. Muscle thickness was defined as the distance between the superficial and deep aponeuroses at each end of the image. The pennation angle was defined as the angle formed between a muscle fascicle and its deep aponeurosis [29]. The measurement sites for the VL and RF were marked and recorded at 36% and 57%, respectively, of the distance from the superior border of the patella to the anterior superior iliac spine [30]. Participants were supine during these measurements. Anatomical landmarks were rechecked at each session before imaging.

### 2.5. Sample Size

The sample size was estimated using GPower software (version 3.1.9.2; Franz Faul, Universität Kiel, Kiel, Germany). The selected effect size was categorized as large (>0.6), which was substantiated by previous and subsequent study markers of oxidative stress conducted specifically on ultramarathon runners [31]. Furthermore, at the level of Type I error (α) 0.05 and Type II error (1 − β) 0.95, the estimated total sample size was 32 volunteers. To account for potential attrition during follow-up (20%), the minimum number of participants was determined to be 39.

### 2.6. Bias

A physiotherapist with >5 years of musculoskeletal ultrasound experience executed the ultrasound protocol. Each measurement site was assessed thrice to evaluate intra-rater reliability, specifically using the intra-class correlation. Intra-class reliability pertains to the consistency or stability of measurements conducted by the same observer [32]. To evaluate inter-rater reliability and mitigate the risk of bias due to potential measurement variability, a secondary assessment was conducted on a sample of 12 subjects. This evaluation focused on specific time points and muscles: pre-laboratory measurement (MT of the rectus femoris), post-race (PA of the vastus lateralis), and post-race (MT of the medial gastrocnemius). The intraclass correlation coefficient (ICC) was calculated using the ICC (3,1) model, as classified by Shrout and Fleiss [32]. This model is suitable when the same raters assess all subjects and are considered fixed effects, estimating the absolute agreement between individual measurements. In both scenarios, the ICC was calculated and categorized as follows: poor (<0.4), moderate (0.4–0.75), and good (>0.75) [33].

### 2.7. Statistical Analysis

Descriptive statistics were computed to characterize the sample. Continuous variables are presented as means ± standard deviation. The data distribution was visually inspected to ensure the appropriate interpretation of the results. To examine the effects of time, condition, and muscle on muscle thickness and pennation angle, linear mixed models (LMMs) were employed. This approach appropriately accounts for the hierarchical structure of the data and repeated measurements within participants. For each dependent variable (MT and PA), fixed effects included time (Pre vs. Post), condition (Laboratory vs. Race), and muscle (rectus femoris, vastus lateralis, and medial gastrocnemius), as well as all interaction terms (time × condition, time × muscle, condition × muscle, and time × condition × muscle). A random intercept for participant ID was included in all models to account for within-subject variability. Random slopes were not included in the final models to avoid overparameterization and potential singular model fits. Models were estimated using restricted maximum likelihood (REML). Statistical inference for fixed effects was conducted using Type III analysis of variance with the Satterthwaite approximation for degrees of freedom. marginal means (EMMs), along with their standard errors and 95% confidence intervals, were calculated to facilitate interpretation and to describe patterns across conditions, time points, and muscles. Model assumptions were assessed through visual inspection of residual plots to evaluate normality and homoscedasticity. All analyses were performed using JASP (version 0.19.1), and statistical significance was set at *p* < 0.05.

## 3. Results

### 3.1. Recruitment, Program Feasibility and Safety: Attendance, Compliance

Within the laboratory conditions, attendance was defined as the number of participants who initiated the experimental protocol, whereas compliance referred to the proportion who completed all required assessments. Of the 40 participants who initiated the laboratory conditions, 36 completed the protocol, resulting in a compliance rate of 90%. Similarly, in the race condition, attendance referred to the number of participants who started the protocol, whereas compliance represented those who completed both pre- and post-assessments. Of the 40 participants who initiated this condition, 29 completed all required measurements, yielding a compliance rate of 72.5%.

### 3.2. Descriptive Analysis

A total of 29 participants were included in the study, each evaluated under two intervention conditions (Laboratory and Race). All available observations were included in the mixed models. The descriptive characteristics of the sample are presented in Table 1. The participants’ mean age was 45.28 ± 7.55 years, average body weight was 68.22 ± 10.93 kg, mean height was 171.67 ± 8.82 cm, and body mass index was 23.02 ± 2.37 kg/m^2^.

### 3.3. Regression Model Pennation Angle

The LMMs demonstrated significant main effects of condition and muscle on the pennation angle, with no significant effect of time detected. Specifically, no significant effect of time was observed (F = 0.908; *p* = 0.341), indicating that the pennation angle did not differ significantly between the pre- and post-intervention measurements. The EMMs showed variable descriptive patterns across muscles and conditions. For example, in the rectus femoris muscle, the pennation angle slightly increased in the laboratory condition from pre- (EMM = 16.433 ± 0.868; 95% CI: 14.732–18.135) to post-intervention (EMM = 16.788 ± 0.868; 95% CI: 15.086–18.490), with a more pronounced increase observed in the race condition (pre-: 14.193 ± 0.899 [12.431–15.955]; post-: 14.959 ± 0.992 [13.015–16.903]). In the vastus lateralis muscle, an increase was observed in laboratory conditions (pre: 17.040 ± 0.868 [15.338–18.742]; post: 18.472 ± 0.883 [16.741–20.203]), whereas a smaller increase was found in race conditions (pre: 14.810 ± 0.899 [13.048–16.572]; post: 15.673 ± 0.992 [13.729–17.617]).

**Figure 1:**
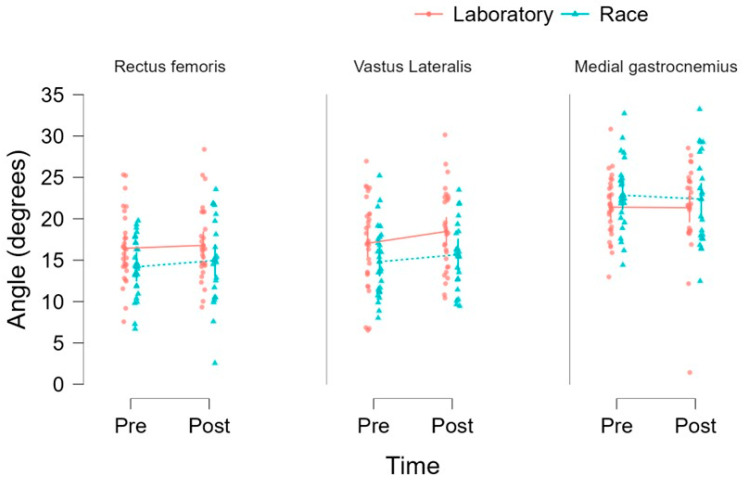
Comparison of muscle angle measurements (degrees) for the rectus femoris, vastus lateralis, and medial gastrocnemius at two time points (Pre and Post). Individual data points are shown for each condition, with red circles representing laboratory measurements and blue triangles representing race measurements. Mean values for each group are connected by lines to illustrate changes over time. Overall, variability is observed within each muscle, with modest differences between conditions and time points.

For the medial gastrocnemius muscle, the pennation angle remained relatively stable in laboratory conditions (pre: 21.401 ± 0.868 [19.699–23.103]; post: 21.342 ± 0.899 [19.580–23.104]) and showed a slight decrease in race conditions (pre: 22.850 ± 0.899 [21.088–24.612]; post: 22.382 ± 0.992 [20.437–24.326]). A significant main effect of muscle was identified (F = 63.062; *p* < 0.001), indicating differences in pennation angle across the muscles analyzed, with higher values observed in the medial gastrocnemius compared to the vastus lateralis and rectus femoris. Additionally, a significant main effect of the condition was found (F = 4.723; *p* = 0.031), indicating differences in pennation angle between the experimental conditions. No significant interactions were observed for time × condition (F = 0.035; *p* = 0.852), time × muscle (F = 0.660; *p* = 0.518), or the three-way interaction (F = 0.092; *p* = 0.912). However, a significant condition × muscle interaction was detected (F = 5.496; *p* = 0.005), indicating that the differences between the conditions varied depending on the muscle analyzed (see Figure 1).

### 3.4. Regression Model Muscle Thickness

The LMMs revealed a significant main effect of muscle on muscle thickness, with no significant effects detected for time, condition, or interaction terms. Specifically, no significant time effect was observed (F = 0.002; *p* = 0.967), indicating that muscle thickness did not significantly change between the pre- and post-intervention measurements. The EMMs showed variable descriptive patterns across muscles and conditions. For example, in the rectus femoris muscle, thickness increased in the laboratory condition from pre-intervention (EMM = 1.723 ± 0.369; 95% CI: 1.000–2.445) to post-intervention (EMM = 2.495 ± 0.369; 95% CI: 1.773–3.218), while remaining relatively stable in the race condition (pre: 2.551 ± 0.382 [1.803–3.299]; post: 2.522 ± 0.421 [1.696–3.348]). In the vastus lateralis muscle, a decrease was observed in the laboratory condition (pre: 3.431 ± 0.369 [2.709–4.154]; post: 2.498 ± 0.369 [1.775–3.220]), with slight reductions also noted in the race condition (pre: 2.650 ± 0.382 [1.901–3.398]; post: 2.459 ± 0.421 [1.634–3.285]).

**Figure 2:**
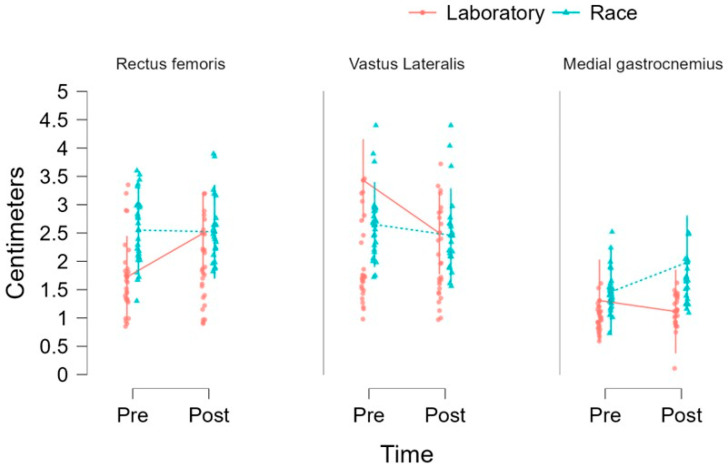
Comparison of muscle thickness (centimeters) for the rectus femoris, vastus lateralis, and medial gastrocnemius at two time points (Pre and Post). Individual data points are presented for each condition, with red circles indicating laboratory measurements and blue triangles indicating race measurements. Group means are connected by lines to illustrate temporal changes. Variability is evident across all muscles, with differing patterns of change between conditions, including slight increases, decreases, or stability depending on the muscle and measurement context.

For the medial gastrocnemius muscle, thickness remained relatively stable in laboratory conditions (pre: 1.307 ± 0.369 [0.585–2.030]; post: 1.114 ± 0.375 [0.379–1.849]) but increased in race conditions (pre: 1.461 ± 0.382 [0.713–2.209]; post: 1.981 ± 0.421 [1.155–2.807]). A significant main effect of the muscle was also identified (F = 12.665; *p* < 0.001), indicating differences in muscle thickness across the muscles analyzed, with overall higher values observed in the vastus lateralis compared to the rectus femoris and medial gastrocnemius. No significant effect of the condition was found (F = 0.668; *p* = 0.415), and no significant interactions were observed for time × condition (F = 0.260; *p* = 0.610), time × muscle (F = 1.764; *p* = 0.173), condition × muscle (F = 1.901; *p* = 0.151), or the three-way interaction (F = 1.430; *p* = 0.241), indicating that the observed patterns over time were consistent across conditions and muscles (see Figure 2).

## 4. Discussion

The present study investigated the effects of time, condition, and muscle on muscle thickness and pennation angle. Regarding muscle thickness, no significant changes were observed over time or between conditions, indicating that the intervention did not result in measurable alterations in muscle size within the analyzed timeframe. The absence of significant interaction effects further suggests that these patterns were consistent across muscles and experimental conditions. However, a significant main effect of muscle was identified, reflecting inherent morphological differences, with the vastus lateralis exhibiting greater thickness than the rectus femoris and medial gastrocnemius. Importantly, given the immediate post-exercise assessment, these findings should be interpreted as reflecting acute responses rather than true structural adaptations. In contrast, the analysis of pennation angle revealed a different pattern. Although no significant time-related changes were detected, a significant condition effect was observed, indicating that the pennation angle differed between the laboratory and race settings. Furthermore, the presence of a significant condition × muscle interaction suggests that these differences were muscle-specific. This indicates that certain muscles may respond differently depending on the context in which they are assessed. However, these variations are more likely attributable to acute factors, such as fluid shifts, muscle swelling, or measurement variability, rather than genuine architectural remodeling. Taken together, these findings suggest that whereas muscle thickness appears relatively stable across conditions and time points, the pennation angle may be more sensitive to context-dependent factors, particularly in a muscle-specific manner.

Our findings related to changes in muscle thickness and pennation angle following fatigue partially align with previous literature; however, they should be interpreted with caution given the acute nature of the measurements. While previous research has attributed increases in muscle thickness primarily to sarcomeric adaptations rather than changes in passive muscle–tendon properties [34], the present results showed no significant changes over time or between conditions. This suggests that the observed variations are unlikely to reflect true structural remodeling and are more plausibly explained by acute factors, such as fluid shifts, muscle swelling, or measurement-related variability. Although descriptively, some differences were observed between the laboratory and race conditions, these were not statistically significant, reinforcing the notion that short-term fatigue does not induce measurable hypertrophy. Importantly, participants maintained their regular training routine throughout the study, further supporting that no long-term architectural adaptations would be expected within the analyzed timeframe. In line with previous evidence indicating that sufficient mechanical tension is required to induce meaningful architectural changes [35], the lack of significant differences between conditions may reflect an insufficient or nonspecific stimulus.

The absence of significant changes in muscle thickness, together with the lack of corresponding alterations in maximal strength, suggests that any observed variations are unlikely to reflect physiological hypertrophy. Instead, they may be attributable to technical or postural variability during measurement. Considering the inter-session reliability estimates reported in the literature (ICC 0.85–0.97, SEM 0.5–1.2 mm), changes within the SEM range are more likely to represent measurement errors rather than real muscular changes. This highlights the importance of strictly standardizing participant positioning to distinguish between actual physiological responses and methodological variability. Although strength was measured pre- and post-intervention, no significant changes were observed, reinforcing that post-fatigue differences in muscle thickness are primarily influenced by measurement conditions rather than by functional adaptations. In contrast, the pennation angle exhibited a different pattern, showing significant differences between the conditions and the condition × muscle interaction. While previous studies have described a relationship between muscle size and pennation angle [36], the absence of concurrent changes in muscle thickness in the present study suggests that these findings are unlikely to reflect structural adaptations. Instead, they may be driven by acute, context-dependent factors, such as muscle hydration status, transient swelling, or alterations in muscle tension immediately following fatigue.

The muscle-specific nature of these responses further highlights the complexity of architectural behavior under different physiological conditions, suggesting that the pennation angle may be more sensitive to contextual influences than muscle thickness. Ultrasound remains an invaluable tool for monitoring muscle architecture [37]; however, its sensitivity to methodological factors must be considered. Traditional assessments frequently rely on static imaging protocols conducted at rest, which may not adequately capture dynamic changes in muscle architecture under functional conditions, such as fatigue, loading, or movement. A more functional approach entails conducting real-time ultrasound assessments during or immediately after specific motor tasks under physiological demand [38], although this may also increase measurement variability.

To ensure the accuracy of our architectural measurements, inter-rater reliability was assessed. High reliability was observed for muscle thickness measurements, supporting the validity of ultrasonography in tracking architectural changes under different experimental conditions [39,40]. Conversely, the pennation angle showed greater variability, consistent with previous literature, emphasizing the need for more standardized measurement protocols and automated analysis tools to reduce subjective bias. Specifically, post-race pennation angle measurements of the vastus lateralis exhibited poor inter-rater reliability (ICC = 0.073; 95% CI: −0.257 to 0.389), indicating poor agreement and substantial variability between raters. In contrast, rectus femoris muscle thickness (ICC = 0.859; 95% CI: 0.742–0.925) and medial gastrocnemius muscle thickness (ICC = 0.929; 95% CI: 0.866–0.963) demonstrated good-to-excellent reliability. These findings support the use of ultrasound for assessing muscle thickness, while highlighting the need for caution when interpreting pennation angle data.

Although this study did not directly assess the effects of ambient temperature, the absence of significant changes in muscle thickness across conditions is consistent with previous findings, suggesting that heat does not impair muscle function during prolonged moderate-intensity exercise [41]. However, the differences observed in the pennation angle between the laboratory and race conditions may still reflect contextual influences associated with real-world competition environments. This study had several limitations. The sample size was relatively small, which may limit generalizability and reduce statistical power. Furthermore, external factors, such as hydration status, environmental conditions, and timing of post-exercise measurements, were not fully standardized, which may have influenced the results. Finally, the lower reliability observed for the pennation angle highlights the need for improved standardized protocols and potentially automated or semi-automated image analysis techniques to reduce evaluator-dependent variability. Future research should explore the acute neuromuscular and mechanical mechanisms underlying context-dependent changes in muscle architecture rather than attributing these responses to long-term structural adaptations.

## 5. Conclusions

In conclusion, the present findings indicate that acute architectural muscle responses were largely stable across time and conditions, with no significant changes observed in muscle thickness and no interaction effects supporting differential responses between conditions or muscles. Although descriptive differences were observed, these patterns were not statistically significant and should therefore be interpreted with caution. In contrast, the pennation angle appeared to be more sensitive to contextual factors, as evidenced by the significant effect of the condition and its interaction with the muscle. This suggests that differences between the laboratory and race conditions may vary depending on the specific muscle analyzed, reflecting a muscle-dependent response to contextual or environmental influences. Importantly, given that assessments were conducted immediately post-exercise, the observed variations are more likely to reflect acute responses rather than true structural adaptations. Additionally, some of the differences observed may be attributable to measurement variability or participant positioning, highlighting the importance of standardized assessment procedures in future research. Overall, these findings emphasize the relative stability of muscle thickness under acute fatigue conditions and suggest that the pennation angle may be more responsive to context-dependent and muscle-specific factors. These results underscore the need to interpret muscle architectural changes within the framework of acute physiological responses and methodological considerations, rather than as evidence of long-term structural remodeling.

## Figures and Tables

**Table 1:** Descriptive characteristics and muscle outcomes across conditions.

Variable	Total (*n* = 29)	Laboratory Pre	Laboratory Post	Δ% Lab	Race Pre	Race Post	Δ% Race
Participant characteristics							
Age (years)	45.28 ± 7.55	–	–	–	–	–	–
Height (cm)	171.67 ± 8.82	–	–	–	–	–	–
Weight (kg)	68.22 ± 10.93	–	–	–	–	–	–
BMI (kg/m^2^)	23.02 ± 2.37	–	–	–	–	–	–
Muscle thickness (cm)							
Rectus femoris	–	1.72 ± 0.37	2.50 ± 0.37	+45.1%	2.55 ± 0.38	2.52 ± 0.42	−1.1%
Vastus lateralis	–	3.43 ± 0.37	2.50 ± 0.37	−27.1%	2.65 ± 0.38	2.46 ± 0.42	−7.2%
Medial gastrocnemius	–	1.31 ± 0.37	1.11 ± 0.38	−15.3%	1.46 ± 0.38	1.98 ± 0.42	+35.6%
Pennation angle (°)							
Rectus femoris	–	16.43 ± 0.87	16.79 ± 0.87	+2.2%	14.19 ± 0.90	14.96 ± 0.99	+5.4%
Vastus lateralis	–	17.04 ± 0.87	18.47 ± 0.88	+8.4%	14.81 ± 0.90	15.67 ± 0.99	+5.8%
Medial gastrocnemius	–	21.40 ± 0.87	21.34 ± 0.90	−0.3%	22.85 ± 0.90	22.38 ± 0.99	−2.1%

## Data Availability

The datasets used and/or analyzed in the current study are available from the corresponding author upon reasonable request.

## Abbreviations

The following abbreviations are used in this manuscript:

ANOVA	Analysis of Variance
BMI	Body Mass Index
CI	Confidence Interval
EMMs	Estimated Marginal Means
GM	Medial Gastrocnemius
ICC	Intraclass Correlation Coefficient
ID	Participant Identifier
Lab	Laboratory condition
LMMs	Linear Mixed Models
Race	Real race condition
RA	Rectus Femoris
REML	Restricted Maximum Likelihood
SD	Standard Deviation
SE	Standard Error
SEM	Standard Error of Measurement
VL	Vastus Lateralis
